# State of the Evidence for Use of Psychotropic Medications in School-Age Youth

**DOI:** 10.3390/children10091454

**Published:** 2023-08-26

**Authors:** Jeffrey D. Shahidullah, Holly Roberts, John Parkhurst, Rachel Ballard, Jennifer A. Mautone, John S. Carlson

**Affiliations:** 1 Department of Psychiatry & Behavioral Sciences, Dell Medical School, The University of Texas at Austin, Austin, TX 78712, USA; jeff.shahidullah@austin.utexas.edu; 2 Department of Psychology, Munroe-Meyer Institute, University of Nebraska Medical Center, Omaha, NE 68198, USA; hroberts@unmc.edu; 3 Pritzker Department of Psychiatry & Behavioral Sciences, Ann & Robert H. Lurie Children’s Hospital of Chicago, Northwestern University Feinberg School of Medicine, Chicago, IL 60611, USA; jtparkhurst@luriechildrens.org (J.P.); rballard@luriechildrens.org (R.B.); 4 Department of Child and Adolescent Psychiatry and Behavioral Sciences, Children’s Hospital of Philadelphia, Department of Psychiatry, Perelman School of Medicine, University of Pennsylvania, Philadelphia, PA 19146, USA; mautone@chop.edu; 5 Department of Counseling, Educational Psychology, & Special Education, Michigan State University, East Lansing, MI 48824, USA

**Keywords:** mental health, psychotropic medications, school age youth, pediatric psychopharmacology

## Abstract

Psychotropic medications are commonly prescribed to school-aged youth for the management of mental health concerns. This paper describes the current state of evidence for psychotropic medications in school-aged youth. More specifically, the following sections summarize relevant medication research trials and practice parameters pertaining to psychotropic medication prescribing as well as the specific medications indicated for a range of commonly presenting disorders and symptom clusters in school-aged youth. For each of these disorders and symptom clusters, key findings pertaining to the current state of science and practice are highlighted for the purpose of offering patients, clinicians, researchers, and policymakers with nuanced considerations for the role of psychopharmacology within the context of a larger “whole-child” approach to care that relies on the collaboration of providers and services across systems of care to promote optimal child and family health and wellness. The paper concludes with a discussion about supporting the use of medication treatments in schools, including considerations for ensuring effective family-school-health system collaboration to best meet youth mental health needs.

## 1. Introduction

Childhood mental health problems are prevalent and associated with an increased risk of difficulties at home, school problems, and mental health and health conditions in adulthood. Roughly 20% of youth living in the United States (U.S.) have an identified mental health condition in a given year, while 40% will meet the criteria by age 18 [1]. Youth in families living below 100% of the federal poverty level have an even higher likelihood of experiencing a mental health condition [2]. Among U.S. children and adolescents, anxiety and attention-deficit/hyperactivity disorder (ADHD) are the most prevalent disorders, each affecting approximately one in 11 (9.4–9.8%) youth [3]. In addition, one-fifth (20.9%) of youth have experienced a major depressive episode, 36.7% of U.S. high school students in 2019 reported persistently feeling sad or hopeless in the past year, and 18.8% seriously considered attempting suicide. Mental health concerns in youth have increased since the onset of the COVID-19 pandemic [4,5], with mental health-related emergency department visits increasing by 24% for children ages 5 to 11 and 31% for ages 12 to 17 in the six months after the pandemic’s onset [6].

Evidence-based therapies for managing prevalent mental health conditions, such as anxiety, depression, and ADHD, include both psychosocial and psychopharmacological approaches [7,8,9,10]. Psychotropic medications are commonly recommended in prominent professional practice parameters and treatment guidelines for a range of mental health conditions (e.g., American Academy of Child and Adolescent Psychiatry [AACAP], American Academy of Pediatrics [AAP]). Unfortunately, one-half of children with mental health concerns do not receive appropriate treatment, including psychosocial therapies or psychotropic medication [11]. Underutilization of evidence-based mental health therapies is due to a variety of factors, including service-level (e.g., limited availability of effective clinicians), societal (e.g., bias, discrimination, state/local policy), economic (e.g., insurance status), and individual (e.g., cultural beliefs, stigma, transportation, parental work leave [12]).

Compounding challenges with access to psychotropic treatments in particular, there is a critical lack of child and adolescent psychiatrists, particularly in areas with a high proportion of families with low incomes and post-secondary education rates and in non-urban/-metropolitan areas [13,14]. Although data suggests an increase in the number of available child psychiatrists in the U.S. (child psychiatrists [per 100,000 children] increased by 22% from 2007 to 2016), the rate of growth cannot keep up with increasing mental health needs [13]. Child mental healthcare demands have increased as a function of expanded child health insurance [15], improved screening and detection for mental health concerns [16], and greater public health awareness of mental health conditions, particularly since the COVID-19 pandemic and the national declaration of a “youth mental health crisis” [17].

To keep up with demand, pediatricians and other primary care clinicians are shouldering more of the load in mental healthcare [18,19]. Despite an American Academy of Pediatrics recommendation [20] that pediatricians achieve competence in providing care for anxiety, depression, and ADHD, among other conditions, surveys suggest that pediatricians lack adequate training in this area [21,22,23]. Primary care clinicians report a lack of comfort and confidence in prescribing medications other than stimulants (anti-anxiety, mood stabilizing, and anti-psychotic medications) and although they identify concurrent psychosocial treatment to be very useful, they often consider this type of support to be inaccessible to patients [24].

Management of psychotropic medication by primary care clinicians has more recently been supported by the use of coordinated, collaborative, and integrated care models. Coordinated models allow prescribing clinicians to consult with on-call psychiatric specialists provided across regions or networks to support their management of patients’ psychotropic medication. The longest-running model is the Massachusetts Child Psychiatry Access Project (MCPAP), which has yielded promising results. However, these telephonic psychiatric access programs rely heavily on state funding or grants to support and sustain the model [25]. Collaborative models address the needs of primary care clinicians onsite through ongoing screening for specific high-frequency conditions (e.g., ADHD, anxiety, depression) and protocol-driven decision-making processes in conjunction with onsite behavioral health clinicians or care managers and offsite psychiatric consultations [26,27]. Integrated models embed behavioral health clinicians within the primary care practice setting to support collaborative evaluation and treatment of child and adolescent mental health needs. Psychologists with requisite training have been particularly well-positioned to undertake collaborative medication-related roles with primary care clinicians in (1) supporting diagnostic clarity, (2) providing psychotropic information to families, (3) joining planning and care coordination with primary care clinicians and families, (4) addressing barriers to adherence, and (5) evaluating and monitoring medication effects [23].

Although the use of psychotropic medications to treat youth mental health concerns has increased in general over the last decade, prescribing rates differ depending on a number of geographic, socioeconomic, and demographic factors [28,29,30]. The National Center for Health Statistics (NCHS) [31] reporting on 2019 trends in mental health treatment among children aged 5–17 years found that males (9.8%) were more likely than females (7.0%) to have been treated with medication and that the prevalence of psychotropic prescribing in non-urban areas (11.8%) was higher than in urban areas (7.4%). NCHS data also suggested that White children were more than twice as likely (11.4%) to have taken medication than Latinx (4.7%) and Black (5.6%) children. Although Black and Latinx youth are generally less likely than other racial/ethnic groups to receive psychotropic medications to treat mental health concerns [32,33], Latinx youth are more likely than other groups to receive antipsychotic drugs, which tend to have more metabolic side effects than other medication classes [34]. Additionally, in a study using data from over 50,000 participants in the National Survey of Children’s Health—2016, children in immigrant families were significantly less likely to have received psychotropic medication for a mental health problem [35].

In a retrospective cohort study of children within one state’s Medicaid database, Lohr et al. [36] found that children in foster care received alpha agonists (116 vs. 69 per 1000 children) or antidepressants (225 vs. 176 per 1000) at a higher rate than children who were not in foster care, but received stimulants at a lower rate (403 vs. 638 per 1000) and children in foster care (beta = 1.83, 95% CI = 1.53, 2.13) had a higher incidence of being prescribed medications for multiple drug classes (i.e., polypharmacy) [34]. In a study exploring antipsychotic prescriptions for children aged 5 years and younger enrolled in a state Medicaid program in one U.S. state, Lòpez-De Fede et al. [37] found that 2 in 5 children received antipsychotics for off-label use (a use other than their FDA-approved indication) and 3 in 4 children also received medications from at least one other psychotropic drug class. In a multisite Developmental Behavioral Pediatrics Research Network (DBPNet) study [38] tracking psychotropic medication prescribing to 3–5-year-old children, those on Medicaid were more likely to be prescribed psychotropic medications than those with private insurance (OR: 1.65; 95% CI, 1.29–2.12) and this was particularly true for alpha-2-adrenergic agonists (OR: 2.48; 95% CI, 1.56–3.92) and atypical antipsychotics (OR: 2.57; 95% CI, 1.46–4.55).

The many professional practice parameters that endorse the use of psychotropic medications in treating a range of child mental health conditions clearly dictate that child mental health professionals use these particular therapies in treatment decision-making. However, the decision for patients, families, and clinicians to use these particular therapies remains incredibly nuanced, and sometimes controversial given that these practice parameters can offer broad guidance without accounting for the many intricacies that must be weighed in any given clinical-decision making process, such as (1) the quality of the research literature including methodological rigor, sampling considerations (i.e., heterogeneity and representativeness of multiple forms of diversity in the samples), and understanding of long-term outcomes, (2) the consideration of patient and families attitudes, acceptability, and satisfaction towards medications that may affect adherence issues and continued help-seeking, (3) the degree to which psychotropic medication prescribing in community settings matches the standard of psychotropic medication prescribing in the RCTs/trials in which it was studied, (4) the balance between the positive benefit and adverse/side effects in the context of the “First, Do No Harm” medical ethical mantra, and (5) the degree to which treatment decision-making can be supported through multi-modal, multi-system, multi-setting, and multi-professional processes to support “whole-child” care while emphasizing the balance between safety, efficacy, and patient preference/autonomy.

The purpose of this paper is to describe the current state of evidence for psychotropic medications in school-aged youth. The following sections summarize the relevant medication research trials and practice parameters (see each citation for details about the systematic literature review methods that were utilized to develop each practice parameter) pertaining to psychotropic medication prescribing as well as the specifically indicated medications for a range of commonly presenting disorders and symptom clusters in school-aged youth (ADHD, Major Depressive Disorder and Persistent Depressive Disorder, Anxiety Disorders, Compulsive, Rigid, and Repetitive Behaviors, and Aggression, Irritability, and Self-Injury). Next, for each of these disorders and symptom clusters, key findings from each practice parameter pertaining to the current state of science and practice are highlighted for the purpose of offering patients, school and medical professionals, researchers, and policymakers with nuanced considerations for the role of psychopharmacology within the context of a larger “whole-child” approach to care that relies on the collaboration of providers and services across systems of care to promote optimal child and family health and wellness. To this end, the paper concludes with a discussion about supporting the use of medication treatments in the school setting, including considerations for ensuring effective family-school-health system collaboration to best meet youth mental health needs.

## 2. Commonly Presenting Disorders and Symptom Clusters

### 2.1. ADHD

ADHD is one of the most common chronic neurodevelopmental disorders among children and adolescents, affecting as many as 10% of youth between ages 3 and 17 [1]. An increased focus on the assessment of ADHD symptoms in diverse populations is paramount [39] given recent meta-analytic data indicating significant under-identification of ADHD in Black individuals in the United States [40]. Those diagnosed with ADHD experience impairment in social relationships and/or academic performance beginning in childhood, often continuing into adolescence and adulthood [41].

Current evidence-based ADHD assessment practices focus on collecting neurodevelopmental information associated with context-impairing symptoms of inattention, disorganization, hyperactivity, and/or impulsivity [41]. Three diagnostic categories are primarily considered when assessing ADHD symptoms in children including ADHD, Combined Presentation, Predominantly Inattentive Presentation, and Predominantly Hyperactive-Impulsive Presentation. Subthreshold symptoms may result in a diagnosis of Other Specified ADHD or Unspecified ADHD. An ADHD diagnosis in childhood is associated with poor health outcomes, and a recent study found a twofold risk for premature death by the age of 46 [42]. Clinicians should recognize that the presentation of hyperactive and impulsive symptoms are associated with a range of negative outcomes, including peer rejection, aggression, risky behaviors, and accidental injuries [39]. Data collection and progress monitoring of these associated deleterious developmental outcomes should be an essential component of clinical care and treatment decision-making.

#### 2.1.1. Practice Parameters and Treatment Guidelines for ADHD

Several professional organizations have developed practice parameters and clinical guidelines for the diagnosis and treatment of ADHD including the American Academy of Pediatrics (AAP) [43] and the Society of Developmental and Behavioral Pediatrics (SDBP) [44]. The SDBP guidelines [44] include a focus on complex cases of ADHD and were developed to complement AAP guidelines [43]. Professional guidelines emphasize the chronic, neurobiological etiology of ADHD and the plethora of evidence supporting effective psychosocial and pharmacological treatments [39].

Current treatment guidelines have been heavily influenced by findings from the NIMH-funded Multimodal Treatment of ADHD (MTA) study [10] and the Preschool ADHD Treatment Study (PATS) [45]. The large, multi-site MTA study [10] was designed to evaluate treatments for children ages 7 to 10 with ADHD (*n* = 579) including medication, behavior therapy, a combination of medication and behavior therapy, and routine community care (control group). Combined (i.e., medication and behavior therapy) treatment and medication alone were found to be significantly superior to behavior therapy and routine community care after 14 months. Outcomes for children in the combination treatment group were obtained with medication doses that were 20% lower than the doses of children in the medication-only group. In general, children prescribed medication through the study had more positive outcomes than those who received medication from community providers (i.e., community control group). Notably, community providers tended to prescribe substantially lower doses of medication (i.e., almost half). While behavioral improvements were maintained at 24 months [46], it was noteworthy that no significant differences were noted at 36-month follow-ups [47]. PATS [45] examined medication efficacy for young children (ages 3 to 5.5 years) with ADHD. Study participants received 10 weeks of behavioral therapy prior to being randomly assigned to either the methylphenidate or placebo groups. Methylphenidate significantly improved symptoms although with smaller effect sizes and more side effects compared to the older age range in the MTA study.

Overall, the MTA and PATS studies revealed initial improvements in medication and combined medication and behavior therapy groups with the MTA study indicating that short-term improvements at 14 months did not generalize long-term when assessed at 36 months [48]. Additionally, given short-acting preparations were used in these studies and extended-release preparations are now more widely used, the generalizability of the results remains unclear. It is noteworthy that methodological factors likely resulted in more favorable outcomes for the medication treatment group in the MTA study [49]. Specifically, dosing in the medication group was significantly higher than what would be expected in community samples. Additionally, although stimulants were beneficial as a short-term treatment, the effects of medication are not observed once the medication is terminated which is often the case for children with ADHD across development. However, current treatment recommendations support medication management for children and adolescents with ADHD along with behavior management across home and school.

As a result of the chronic nature of the disorder, professional guidelines encourage a chronic care, life course approach to treatment [43,44,50], with modifications to treatment planning consistent with child development. Also, consistent with a developmental approach to treatment, there are specific practice parameters for preschool-aged children (3 to 5 years), children (6 to 12 years), and adolescents (13 to 17 years). For preschool-aged children, behavioral intervention, specifically behavioral parent training, is considered the first line of treatment, supplemented by medication in cases of ongoing moderate to severe impairment. For children over age 6, FDA-approved medications for ADHD are encouraged, in combination with behavioral intervention (i.e., behavioral parent training and behavioral classroom intervention). Guidelines emphasize the importance of engaging adolescent patients in care planning, whether the treatment plan includes medication and/or behavioral intervention (e.g., evidence-based youth skills training).

#### 2.1.2. Medication Management for ADHD

Treatment guidelines across the leading practice organizations all recommend behaviorally-based interventions as the first-line treatment for preschool children with ADHD. Stimulants are not approved by the FDA for use in preschool children [43]. According to the AACAP and AAP, stimulants are the first-line treatment [10] and most commonly prescribed psychotropic medications for children and adolescents with ADHD [51]. Stimulants include both methylphenidate and amphetamines products and come in a variety of immediate-release/short-acting and extended-release preparations (see Table 1). Collectively, stimulants increase the availability of dopamine and norepinephrine which are neurotransmitters associated with attention. Approximately 75% of children with ADHD experience significant reductions in the primary symptoms of ADHD (i.e., attention, hyperactivity, impulsivity). When a child with ADHD does not have a favorable response to stimulant medications (e.g., side effects, increased anxiety, decreased mood, tics), non-stimulant medications may be prescribed [43]. The FDA has approved two non-stimulant medication classes for the treatment of ADHD. The first non-stimulant class includes two norepinephrine reuptake inhibitors, atomoxetine (Strattera) and viloxazine (Qelbree). The second non-stimulant class of medications includes two selective alpha-2 agonists, extended-release guanfacine (Intuniv) and extended-release clonidine (Kapvay).

The AAP issued a policy statement [52] in 2014 regarding prescribing practices for off-label medication to children. In this statement, the AAP recommended that scientific evidence rather than label indication serve as the gold standard for practitioners making therapeutic decisions for patients. There are some off-label medications for children with ADHD including bupropion, selective serotonin reuptake inhibitors (SSRIs), and atypical neuroleptic agents (e.g., risperidone and aripiprazole) with limited evidence supporting use for the treatment of ADHD. Bupropion (Wellbutrin) is registered as a unique antidepressant and is thought to have an indirect mixed agonist effect on dopamine and norepinephrine and has also been used with some success to treat adults with ADHD [53]. However, a systematic review of the effectiveness of bupropion revealed the quality of evidence to be low and includes adult samples [54].

Side effect management is among the most significant challenges with children who are managed with stimulant medication with loss of appetite (anorexia) and difficulty falling asleep (insomnia) being the most commonly reported [55]. Additional side effects include dizziness, irritability, or moodiness while taking medication, growth problems (e.g., concerns with height and weight) [56], and heart symptoms (e.g., pounding, almost passing out, chest pain) [57].

### 2.2. Major Depressive Disorder and Persistent Depressive Disorders

Child and adolescent depressive disorders and associated suicidality have a profound psychosocial impact on schools, families, and communities. Meta-analytic methods estimate the global pooled prevalence of elevated depression in school-aged children to be one in four (25.2%) [5]. Of those with severe depression, about one in three experience suicidality [58]. Death by suicide for 10–19-year-olds occurs at a rate of approximately 7 in 100,000 [1], making it the third leading cause of death in this age group.

Current evidence-based depression assessment practices focus on the collection of information associated with the following depressive disorders: Major Depressive Disorder (MDD: Single and Recurrent), Persistent Depressive Disorder (PDD: Dysthymia), Disruptive Mood Dysregulation Disorder (DMDD), Premenstrual Dysmorphic Disorder, Mood Disorder Due to Another Medical Condition, Substance/Medication-Induced Disorder, Other Specified Depressive Disorder, and Unspecified Depressive Disorder [41]. Major Depressive Disorder and Persistent Depressive Disorder are the most commonly diagnosed depressive disorders occurring in about 15% of children and the focus of this section [1]. The most common diagnostic feature of depressive disorders is the presence of persistent sad, empty, or irritable mood. Frequent episodes of behavioral and emotional dysregulation, feelings of hopelessness, low energy, suicidal thoughts, and/or physiological changes such as changes in sleep patterns or appetite may also be present. These symptoms negatively impact one’s functioning at home, school, and/or the community. Symptoms can be experienced on a continuum and individualized assessment utilizing a developmental lens on differential diagnosis and treatment planning is especially important within school-aged populations [59].

#### 2.2.1. Practice Parameters and Treatment Guidelines for MDD and PDD

Employing evidence-based mental health practices to minimize the impact of depressive symptoms on children’s functioning and, in some cases, mortality, is critical. Depressive disorders are believed to be significantly underdiagnosed by mental health professionals creating the need for accurate screening and comprehensive assessment practices [60]. An independent panel of national experts in evidence-based medicine recently recommended that screening for MDD within primary care is necessary to help facilitate early intervention [61].

To improve the quality of behavioral healthcare provided to children and adolescents with depressive disorders, numerous behavioral health professional organizations [i.e., child psychiatry [62,63], pediatrics/primary care [64,65], and psychology [59] have developed assessment and treatment practice guidelines to assist clinicians. Recently published guidelines for the assessment and treatment of depression [63] highlight the following essential biopsychosocial data to be gathered within a thorough diagnostic evaluation: (a) symptom identification including frequency, severity, onset, and duration of each and in combination if co-occurring symptom patterns are evident, (b) the impact and dysfunction associated with symptoms, (c) areas of developmental differences or delays compared to peers, (d) any related somatic concerns, and (e) etiological considerations and other factors either negatively or positively impacting symptom presentation. Gathering information about the child and family’s values, beliefs, and attitudes regarding both symptoms and possible treatment approaches is essential to promote an effective treatment plan to match the unique needs and context of each child. Treatment guidelines for MDD and PDD developed an extensive review of the latest evidence-base [63] including the following:

1. Cognitive-behavioral therapy (CBT) and interpersonal therapy should be offered to school-aged youth with MDD or PDD, as a first-line intervention.
2. Selective serotonin reuptake inhibitor medication (except paroxetine), preferably fluoxetine, should be offered to school-aged youth diagnosed with moderate to severe MDD.
3. Combined treatments (cognitive-behavioral therapy plus fluoxetine) should be offered to school-aged youth diagnosed with MDD, preferably within the same system of care.
4. Continued fluoxetine alone or cognitive-behavioral therapy plus continued fluoxetine should be offered to school-aged youth who respond favorably to acute fluoxetine treatment, in order to prevent MDD relapse/recurrence.

In sum, evidence-based psychosocial treatments (i.e., CBT, interpersonal therapy) alone or combined with SSRI medications should be utilized to treat school-aged youth diagnosed with MDD or PDD. In addition, maintenance of these treatments can help to prevent depression symptom relapse and recurrence. Noteworthy is the need for an accurate diagnosis based on multi-method and multi-informant biopsychosocial data prior to implementing these treatment guidelines.

#### 2.2.2. Medication Management for MDD and PDD

Evidence from a review of clinical trials demonstrates the benefit of antidepressants as a positive response to antidepressants is seen in about 50–70% of those treated, though at rates only about 10–15% higher (this includes all SSRI trials including industry-funded and NIH-funded trials) than those receiving placebo [7]. Of the diverse medications investigated with rigorous randomized controlled studies, fluoxetine has the most evidence for its use to reduce moderate to severe depressive symptoms in the adolescent population [66]. Current primary care treatment guidelines for mild depression include active support and symptom monitoring by clinicians as some mood dysregulation is a normal part of adolescent development [7]. For moderate to severe cases, active monitoring must switch immediately to an active, comprehensive, actionable treatment approach according to these guidelines. Psychotherapy and psychopharmacological treatment are recommended in combination with crisis intervention, family support, skill building, and mental health consultation. A multitude of approaches are essential knowing that antidepressant medications have not been found to improve life functioning measures, other than measures of depressive symptoms [67]. Moderate and severe depressive cases also warrant a close examination of parallel substance or drug use. Finally, if symptom improvement is not observed after 6 to 8 weeks of treatment, a reexamination of diagnosis and a potential modification of treatment planning is recommended within these treatment guidelines directed at primary care professionals.

The most common side effects of SSRIs [63,64] include gastrointestinal symptoms (e.g., nausea, diarrhea), behavioral activation (e.g., restlessness, impulsivity), headaches, and less often, fatigue. Other rare side effects include activation of mania/hypomania and increased suicidal ideation. The most intolerable antidepressant medications are duloxetine, venlafaxine, and paroxetine; highlighting the need for close monitoring of adverse events within school-aged populations being prescribed this class of medications [7]. Specifically, virtual or in-person data gathering on symptoms worsening, suicidality, or any other unusual behavioral health changes is necessary.

### 2.3. Anxiety Disorders

Anxiety disorders represent the most common mental health conditions across the world [68]. Anxiety disorders have an early age of onset and are associated with functional impairment and symptomatic distress [69]. Additionally, anxious youth are at increased risk for developing mood disorders, substance use disorders, suicidal behavior, and significant psychosocial impairment (e.g., educational underachievement, adult economic disadvantage) [70].

Anxiety disorders encompass Separation Anxiety Disorders, Specific Phobias, Generalized Anxiety Disorder, Social Anxiety Disorder, and Panic Disorder. Anxiety disorders tend to present at predictable times throughout childhood. Separation Anxiety typically has an earlier age of onset than social anxiety or panic disorder, which more typically emerge in adolescence and young adulthood [71,72]. Anxiety disorders are frequently comorbid, share similar neurobiology [73] and treatment responses [72]. Three anxiety disorders—Generalized Anxiety, Separation Anxiety, and Social Anxiety—are known as the “pediatric anxiety triad” and have received the most attention in randomized control trials of treatment of anxiety disorders.

#### 2.3.1. Practice Parameters and Treatment Guidelines for Anxiety Disorders

The most recent American Academy of Child and Adolescent Psychiatry [74] practice parameters on the treatment of anxiety disorders in children and adolescents make the following recommendations:

1. Cognitive Behavioral Therapy should be offered to patients 6–18 with separation anxiety, social anxiety, generalized anxiety, specific phobia, and panic disorder.
2. SSRIs may be offered to patients 6 to 18 years old with social anxiety, generalized anxiety, separation anxiety, or panic disorder.
3. Combination treatment (CBT and an SSRI) could be offered preferentially over CBT alone or an SSRI alone to patients 6 to 18 years old with social anxiety, generalized anxiety, separation anxiety, or panic disorder.
4. Serotonin norepinephrine reuptake inhibitors (SNRIs) could be offered to patients 6 to 18 years old with social anxiety, generalized anxiety, separation anxiety, or panic disorder.

There is robust evidence for the efficacy and safety of both CBT and medication treatments for pediatric anxiety disorders [75,76,77]. Psychotherapy is a first-line treatment for anxiety disorders. There is evidence for the use of CBT [78] and other therapies such as Acceptance and Commitment Therapy [79] and mindfulness-based therapies [80] that share common treatment principles with CBT.

#### 2.3.2. Medication Management for Anxiety Disorders

While medication may be used without therapy, often medication is employed in addition to CBT. This is commonly referred to as combination treatment. SSRIs are the first-line medications used for anxiety disorders. SSRIs inhibit serotonin reuptake and, in some cases, inhibit dopamine and norepinephrine reuptake. SSRIs that have shown efficacy with pediatric populations in randomized controlled trials include, fluoxetine (trade name, Prozac) [81], sertraline (trade name Zoloft) [82], and escitalopram (trade name Lexapro) [83]. Table 2 for the most common medications used in the treatment of pediatric anxiety.

The most common side effects of SSRIs include gastrointestinal symptoms (e.g., nausea, diarrhea, lack of appetite), physical activation (e.g., restlessness, insomnia, impulsivity), headaches, and less often, fatigue. Other rare side effects include activation of mania/hypomania and increased suicidal ideation. SSRIs, when initiated, tend to require 4–6 weeks to assess effectiveness. Treatment response to SSRIs is logarithmic, with improvement occurring quickly during the first 8 weeks of treatment and plateauing at 24 weeks [84,85]. This week-over-week improvement appears to be accelerated when medication is combined with psychotherapy [86].

SNRIs are considered a secondary medication option to treat anxiety disorders in youth [85]. Two SNRIs have been found effective in randomized controlled trials for pediatric anxiety disorders: duloxetine (trade name Cymbalta) [87] and venlafaxine (trade name Effexor) [88]. SNRIs have an adverse effect profile similar to that of SSRIs, although they may be slightly less likely to cause activation [85]. Additionally, venlafaxine may increase treatment onset suicidality [66,89].

To date, there are no head-to-head studies of SSRIs and SNRIs in youth with anxiety disorders. Three meta-analyses have looked at outcome response in anxiety for SSRIs and SNRI-evaluated duloxetine, fluoxetine, fluvoxamine, paroxetine, sertraline, and venlafaxine, demonstrating antidepressant superiority relative to placebo with a pooled effect size (Cohen’s *d*) estimate of 0.62 (95% CI: 0.34–0.89; *p* = 0.009). Locher et al. [76] meta-analytically evaluated SSRIs and SNRIs in children and adolescents with anxiety disorders. Locher et al. [76] noted increased remission with SSRIs (relative risk, 2.04; 95% CI: 1.37–3.04) and response (relative risk, 1.96; 95% CI: 1.60–2.40) in a sample including 7700 pediatric patients. According to studies of antidepressants in pediatric patients with anxiety disorders, SSRIs are associated with earlier improvement compared to SNRIs. Benzodiazepines, such as Xanax, did not meta-analytically reduce anxiety [75] and are not recommended for the treatment of anxiety disorders in youth.

The combination of CBT and SSRIs appears to have the strongest impact on the remission of youth anxiety disorders. The NIH-funded Child/Adolescent Anxiety Multimodal Study (CAMS) (*n* = 488) compared placebo, sertraline only, CBT only, and sertraline + CBT (COMB) over 12 weeks. The three treatments, COMB, CBT, and sertraline only, were all more effective than placebo. COMB was more effective than CBT or sertraline alone. Remission rates for COMB were superior to CBT (66% vs. 35%) after 12 weeks of treatment [90]. Youth in CBT at 24 weeks saw the remission rates narrow (65% vs. 45%) [84]. Additionally, relative to either monotherapy, the probability of response for both CBT and CBT + sertraline increased week-over-week during the acute 12-week treatment period, whereas the probability of achieving additional benefit plateaus by week 4 in patients receiving placebo and week 8 in patients receiving sertraline [91]. Given current evidence, it appears likely that youth with severe anxiety disorders would benefit from combination treatment for anxiety.

### 2.4. Compulsive, Rigid, and Repetitive Behaviors

The symptom cluster of compulsive, rigid, and repetitive behaviors represent behaviors that a person feels the urge to do and can often be in response to an obsessive thought, such as in obsessive compulsive disorder (OCD). This symptom cluster also includes conditions such as trichotillomania whereby someone struggles to resist the urge to pull out their hair. Rigid and repetitive behaviors are often symptoms of neurodevelopmental concerns such as autism and may include repetitively flapping arms, spending hours lining up toys, or struggling to tolerate changes in routine.

#### 2.4.1. Practice Parameters and Treatment Guidelines for Compulsive, Rigid, and Repetitive Behaviors

CBT incorporating Exposure and Response Prevention (ERP) is the first-line treatment for mild to moderate OCD [92]. For moderate to severe OCD, the combination of CBT with a SSRI is appropriate therapy. The AACAP [93] practice parameters state that pharmacologic treatment may also be appropriate in milder OCD cases in settings where high-quality CBT is not available, or when there is comorbid anxiety or depression.

The seminal pediatric OCD study was the NIMH-funded Pediatric OCD Treatment (POTS) 2004 study [93] that randomized 112 patients to sertraline, high-quality CBT with ERP, or the combination of sertraline and CBT. The effect sizes for combined treatment, CBT alone, and sertraline were 1.4, 0.97, and 0.67, respectively. Other randomized controlled trials of medication in pediatric OCD include the SSRIs fluvoxamine, fluoxetine, and paroxetine, and the tricyclic antidepressant clomipramine [94].

Skin picking and trichotillomania are classified as obsessive-compulsive related disorders in DSM-5. Both disorders often have onset during childhood or adolescence. Behavioral therapies including Habit Reversal Therapy are superior to pharmacologic treatments and are considered first-line treatment for both disorders [95].

#### 2.4.2. Medication Management for Compulsive, Rigid, and Repetitive Behaviors

SSRIs such as fluoxetine and sertraline are often the first medications considered for the treatment of pediatric OCD and are generally well tolerated [76]. Clomipramine was the first medication FDA approved to treat pediatric OCD but is now generally reserved for refractory cases as its use requires monitoring of heart rate, blood pressure, and electrocardiogram.

Small placebo-controlled trials have demonstrated the efficacy of fluoxetine, citalopram, and lamotrigine in adults with the skin-picking disorder [96]. A randomized controlled trial of N-acetylcysteine (NAC) found a 47% response rate in adults with skin picking disorder compared to a 19% response in those treated with a placebo [97]. A more extensive range of pharmacologic treatments including SSRIs, tricyclics, antipsychotics, opioid antagonists, and NAC has been explored in the treatment of trichotillomania in adults, mostly in small trials, with negative or low-certainty positive results [98]. A randomized controlled trial of NAC in 50 adults resulted in decreased hair pulling compared with placebo; a similar study in 39 adolescents showed no difference between NAC and placebo [99,100]. NAC was well-tolerated in both studies. Based on limited evidence for pharmacologic treatment of skin picking or trichotillomania in children or adolescents, there may be a role for treatment with an SSRI, especially if there is comorbid anxiety or OCD. NAC may be an appropriate treatment option given its safety and tolerability, and relatively promising results in adults with both disorders.

### 2.5. Aggression, Irritability, and Self-Injury

Aggression, irritability, and self-injury are symptoms that may be associated with essentially any psychiatric disorder, and that also present in the absence of psychiatric disorder. The first step in addressing aggression, irritability, or self-injury is to explore the vast array of factors that may underlie the behaviors. These may include physical illness, discomfort, pain, boredom, frustration, a mismatch between external expectations and a child’s capacity, communication deficits, bullying, and other modifiable factors. Aggression, irritability, and self-injury may also occur in the setting of psychiatric disorders. In these cases, assertive treatment of the psychiatric disorder is the first step in treating the behavioral symptoms.

#### 2.5.1. Practice Parameters and Treatment Guidelines for Aggression, Irritability, and Self-Injury

Randomized controlled trials of medications targeting aggression, irritability, and self-injury have focused primarily on pediatric populations with ADHD and/or autism spectrum disorder (ASD) or with other genetic or neurodevelopmental disorders such as Prader-Willi Syndrome. Randomized controlled trials in children and adolescents with ADHD have demonstrated that psychostimulants reduce oppositional and aggressive behaviors as well as core ADHD symptoms [101].

#### 2.5.2. Medication Management for Aggression, Irritability, and Self-Injury

Blader et al. [102] planned a stepped treatment trial for ADHD and aggressive behavior that began with an open stimulant optimization phase before adding adjunctive treatments (risperidone, divalproex, or placebo). Of the 151 youth who completed the stimulant optimization phase, 63% had no residual aggression and so did not move to randomization to adjunctive treatments. This study reinforces the utility of maximizing ADHD and oppositional symptom control with stimulant medications prior to considering other pharmacologic treatment options [102].

Alpha agonists including clonidine and guanfacine, in immediate- and extended-release forms, are second-line or adjunctive treatments for ADHD in children and adolescents. Use of the immediate-release forms is limited by sedation and the need for multiple daily doses. Placebo-controlled trials of clonidine in children with ADHD, some of whom had comorbid oppositional defiant disorder (ODD) or conduct disorder (CD), have been either negative regarding oppositional behavior or conduct outcomes or been positive with a lower limit of 95% confidence interval approaching zero [103]. We found no studies assessing extended-release clonidine in the treatment of oppositional behaviors in children with or without ADHD. Four placebo-controlled trials of guanfacine extended-release in children with ADHD and oppositional behavior with or without comorbid ODD have been conducted. An analysis of these 4 studies found effect sizes ranging from 0.688 to 0.963 among children with ODD and from 0.598 to 0.842 among children without ODD [104].

Alpha agonists have been assessed for the reduction in aggression, irritability, and self-harm in children and adolescents with ASD. Guanfacine extended-release was compared to a placebo in reducing oppositional behavior in 62 subjects with ASD and ADHD symptoms. Parent-reported oppositional behavior decreased by 44% in the active medication group vs. 12% in the placebo group [105].

Other medications that have been evaluated in the treatment of oppositional and aggressive behaviors in children and adolescents with ADHD include risperidone and divalproex. In the Blader et al. study [102], both risperidone and divalproex were superior to placebo in decreasing residual aggressive behavior in children who had gone through stimulant optimization. The numbers of randomized patients were small, the divalproex result approached non-significance and the children who were randomized to adjunctive treatments remained more symptomatic than those who remitted with stimulants only.

The atypical antipsychotics risperidone and aripiprazole have FDA indications for the treatment of aggression, irritability, and self-injury in children aged 5 years and older (6 years and older for aripiprazole) with autism spectrum disorder. The FDA indications were based on a series of positive randomized controlled trials for both drugs [106,107,108,109,110]. The seminal study supporting the efficacy of risperidone in ASD was the Research Units in Pediatric Psychopharmacology (RUPP) study which randomized 101 subjects aged 5–17 years to 8 weeks of risperidone (1.8 = 0.7 mg/day) or placebo [108]. The risperidone-treated subjects had a 57% reduction in irritability score compared with 14% of the placebo group. A 4-month open-label extension of the study demonstrated a stable response and a mean weight gain of 5.1 kg. This was followed by a placebo-substitution risperidone withdrawal phase. The relapse rate in the placebo-substitution group were 62.5% and 12.5% in the risperidone continuation group [111]. Trials of other atypical antipsychotics in treating aggression, irritability, or self-injury in children and adolescents with ASD have not shown superiority over placebo.

The atypical antipsychotics as a class have increasingly been used off-label to treat aggressive behavior in children without ASD. This trend increased rapidly through the 1990′s, leveled off in the 2000′s, but persists particularly in younger children, children in foster care, children with public insurance, and racial minority children [112,113,114,115,116]. Atypical antipsychotics as a class are associated with adverse metabolic effects, including increased appetite, weight gain, elevations in blood sugar and lipids, and dystonic reactions. Because of this high side effect burden, off-label antipsychotic prescribing to children to achieve behavioral control should be temporary, regularly reviewed, and adjunctive to other behavioral improvement strategies.

## 3. Considerations for Supporting Uptake of Medications within School Settings

Approximately half (42.8%) of all children with identified mental health concerns take psychotropic medications to treat these conditions [117,118]. With increases in children who take medication is the demand for school personnel to assist in addressing mental health concerns in the schools. Although decisions to use psychotropic medications are ultimately guided by a child’s family and medical team, effective interdisciplinary collaboration between the child, the child’s family, the school, and the medical team can optimize the implementation and coordination of medical and psychosocial interventions in practice guidelines. The medical team is responsible for monitoring psychotropic medication; however, they cannot monitor the effects of medication on learning and behavior in the classroom (schools, medical providers, and communities are encouraged to partner and develop policies and guidelines supporting students on medications [118]). However, practice guidelines are not always clear and barriers may be present including diagnosis and treatment practices of the medical provider, state/federal legislation, school district policies, and cooperation of all parties (schools, parents, prescribing providers) to work together.

The scientific literature and professional organizations provide practice guidance for treating youth with common mental health conditions. Clinical practice guidelines are intended to help guide practice. However, the publication and dissemination of practice guidelines for treating mental health concerns in children do not always result in the implementation of their use. The effectiveness of improving practice has been debatable for a variety of reasons [119,120] including lack of evidence for improving practice, lack of evidence for providers implementing practice guidelines, and lack of clarity with how or which guidelines to implement and the order of implementation [121]. Practice guidelines often involve randomized control trials (RCTs) that are outdated or have methodological flaws [119]. Additionally, some guidelines, such as ADHD practice guidelines may lack considerations for equitable access to treatment for ADHD especially for Black children, girls, and children from low-income families [122]. Additionally, administration and monitoring of psychotropic medications is complex because more children take psychotropic medication and many of these medications are considered off-label [123]. It is estimated that up to 40% of individuals on medications receive treatment that is not based on organization guidelines or the available scientific evidence [124].

Although practice parameters provide guidance with aspects of managing plans for children with certain educational eligibility and/or medical diagnoses and/or administering medications to children at school, district policies and local, state, and federal law may dictate the ability to apply or synthesize best practices between school and outside medical professionals. There may be district and/or state or federal laws that prohibit school involvement with referral or consultation regarding psychoactive medications [125]. Section 300.174 “Prohibition on mandatory medication” of the Individuals with Disabilities Education Act (IDEA) prohibits state personnel from requiring parents to obtain prescription medications for a child attending school. This same section states that this prohibition does not preclude teachers and school personnel from consulting or sharing classroom observations with parents or guardians or the need for special education or related services. As of 2014, only 11 states had a medication policy that addressed psychotropic medications in schools [123]. Most state policies reflected statements precluding school personnel from recommending psychotropic medication for students. Additionally, Texas was the only state that provided specific legislation regarding policy for the administration of psychotropic medication in schools. States need to develop guidelines that address individual planning for the student, methods for communicating information on benefits and side effects with relevant professionals included in the child’s care, procedures for storage, administration, and monitoring of medication especially when a change in medication has been initiated.

Carlson and Shahidullah [125] recommend that certain assumptions may need to be met in order best guide evaluating the effects of psychotropic medications in schools including (a) a comprehensive evaluation, diagnosis, and treatment plan by a qualified health professional, (b) behaviorally-based interventions have been developed at home and school and that the timing and order of interventions are ultimately the choices of the family, (c) families receive psychoeducation regarding all empirically supported treatments, (d) parents allowed to make the decision to seek medication treatment for their child without influence or pressure from the school, (e) parents’ consent to the sharing of information between the school and medical team and (f) release of information is secured to ensure an understanding of open lines of communication between parents, the school and medical providers.

Another consideration includes providing educational opportunities for school personnel to learn about psychotropic medications. Ryan et al. [118] indicate that given the high prevalence of youth taking psychotropic medications at school, educators have basic knowledge and overview of common psychotropic medications. Many schools do not have full-time nurses available with knowledge and training in psychotropic medications. At times, an unlicensed assistive school team member may be administering medications in the school. Special educators who manage the academic and behavioral success of students indicate almost ubiquitously (95%) that they lack background and knowledge in psychotropic medications and desire additional training [126].

Foy and Earls [122] emphasize the importance of establishing a school-community plan for addressing barriers from identification through treatment. They recommend utilizing practice resources to help with educating and providing school professionals with educational and screening tools, for instance, that will help to achieve a collaborative plan for supporting children. Although developed specifically for youth with ADHD, Foy and Earls’ recommendations for school-physician collaborative plans provide a useful framework to apply to school-age youth with other mental health conditions such as depression, anxiety, and aggressive behavior (see Table 3 for recommendations by Foy & Earl on collaborative school-physician-community plan for ADHD that are applicable to the other child mental health conditions reviewed). The use of a 504 plan or an individualized education plan (IEP) may be needed to ensure a child who is eligible for these plans has documented access to services and resources in the schools and with professionals outside of the school.

## 4. Conclusions

The current state of the psychotropic medication literature for school-aged children has grown substantially in the past 30 years. Prescribing psychotropic medications to school-aged youth for a range of mental health concerns can greatly reduce symptoms and improve functioning across school, home, and social settings. However, these treatments provide symptom relief, but not cures. Moreover, the literature is still growing, and we are far from having empirical justification for all prescribing decisions. Further, inequities in differential access to these therapies and prescribing and monitoring practices clearly necessitate careful consideration of the tradeoff between risks and benefits, particularly for marginalized and vulnerable populations. Finally, it is important to note that a review of the impact of supplements (e.g., food, vitamin, herbal) and over-the-counter medications on child psychiatric conditions was outside the scope of this paper. Though, it must be recognized that none of those non-psychotropic medications have been recognized as efficacious within any of the professional practice parameters reviewed.

Decision-making around medication prescribing is incredibly nuanced and should be informed by considerations such as: (1) the quality of the research literature including methodological rigor, sampling considerations (i.e., heterogeneity and representativeness of multiple forms of diversity in the samples), and understanding of long-term outcomes, (2) the consideration of patient and families’ attitudes, acceptability, and satisfaction towards medication (including cultural and/or religious factors) that may affect adherence issues and continued help-seeking, (3) the degree to which psychotropic medication prescribing in community settings matches the standard of prescribing in the RCTs/trials in which it was studied, (4) the balance between the positive benefit and adverse/side effects in the context of the “First, Do No Harm” medical ethical mantra, and (5) the degree to which treatment decision-making can be supported through multi-modal, multi-system, multi-setting, and multi-professional processes to support “whole-child” care while emphasizing the balance between safety, efficacy, and patient preference and autonomy.

## Figures and Tables

**Table 1:** First-line Medications for the Treatment of ADHD.

Targeted Disorder (Class)	Drug Name	Formulation	Expected Duration of Action
ADHD (Methylphenidate)	Adhanisa XR	Extended-release capsule	Up to 16 h
	Aptensio XR	Extended-release capsule	12 h
	Azstarys XR	Extended-release capsule	12 h
	Concerta	Extended-release tablet	10–12 h
	Contempla XR-ODT	Extended-release orally disintegrating tablet	12 h
	Daytrana	Transdermal patch	6–16 h
	Focalin (dexmethylphenidate)	Short-acting immediate release tablet	4–5 h
	Focalin XR (dexmethylphenidate)	Extended-release capsule	10–12 h
	Jornay PM	Delayed release—Extended-release tablet	12 h
	Metadate CD	Extended-release capsule	6–8 h
	Methylphenidate HCl	Short acting/immediate release chewable tablet	3–4 h
	Methylin Liquid IR	Short Acting Oral solution	3–4 h
	Quillivant XR	Extended-release oral suspension	12 h
	Quillichew ER	Extended-release chewable tablet	8 h
	Ritalin IR	Short-acting, immediate release tablet	3–4 h
	Ritalin LA	Extended-release capsule	8 h
ADHD (Amphetamine)	Adderall	Short-acting, immediate release tablet	6 h
	Adderall XR	Extended release tablet	12 h
	Adzenys XR	Extended release capsule	12 h
	Adzenys ER	Extended release oral solution	10–12 h
	Dexedrine spansule	Spansules	6 h
	Dexedrine tablets	Short-acting capsules	3–5 h
	Dyanavel XR	Oral solution	13 h
	Evekeo ODT	Short-acting tablets	3–5 h
	Mydayis	Extended release capsules	Up to 16 h
	ProCentra	Short-acting tablets	4–5 h
	Vyvanse	Extended release capsules	12–14 h
	Vyvanse	Long-acting chewable	12 h
	Zenzedi	Short-acting tablet	4–5 h

**Table 2:** First line medications for the treatment of Major Depressive Disorder and Persistent Depressive Disorders, Anxiety Disorders, Compulsive, Rigid, and Repetitive Behaviors, and Aggression, Irritability, and Self-Injury.

Generic/Trade Name	Class	FDA Approvals	Starting/Max Dose	Generally Effective Dose
Sertraline/Zoloft	SSRI	OCD ages ≥ 6	25–200 mg	50–150 mg
Fluoxetine/Prozac	SSRI	OCD & Major Depression ages ≥ 7	10–60 mg	20–40 mg
Escitalopram/Lexapro	SSRI	Major Depression ages ≥ 12	5–20 mg	10–15 mg
Duloxetine/Cymbalta	SNRI	Generalized Anxiety ages ≥ 7	30–60 mg	30–60 mg
Venlafaxine/Effexor	SNRI	No pediatric approvals	37.5–375 mg	75–225 mg

**Table 3:** Recommendations for School-Physician-Community Treatment Planning Collaboration for School-Age Youth Experiencing Mental Health Disorders.

Achieve consensus on the role of school personnel regarding the collection of data Use pediatric office visits for to identify children with academic or behavior problems Refer identified children to the contact person at the child’s school who request information on the collaborative consensus plan Designate a contact person at each school to share student information with Review data from the school and incorporate it into the clinical assessment Reinforce the need and type of treatment with parents and schools and considerations for additional accommodations and modifications in the school (e.g., 504 plan and/or IEP) Referral plan to mental health provider when comorbid concerns are identified Develop a system of communication to share diagnostic, treatment, management information between school, physicians, parents Receive parent and teacher follow-up reports to assist in medication management Maintain communication with school especially during transitions (e.g., beginning of the school year, changes in schools, changes in family)

Note. These recommendations were adapted from Foy and Earls [122].

## Data Availability

Not applicable.
